# Exploiting Illumina Sequencing for the Development of 95 Novel Polymorphic EST-SSR Markers in Common Vetch (*Vicia sativa* subsp. *sativa*)

**DOI:** 10.3390/molecules19055777

**Published:** 2014-05-05

**Authors:** Zhipeng Liu, Peng Liu, Dong Luo, Wenxian Liu, Yanrong Wang

**Affiliations:** State Key Laboratory of Grassland Agro-ecosystems, School of Pastoral Agricultural Science and Technology, Lanzhou University, Lanzhou 730020, China

**Keywords:** EST-SSRs, *Vicia sativa* subsp*. sativa*, genetic diversity, Illumina sequencing

## Abstract

The common vetch (*Vicia sativa* subsp. *sativa*), a self-pollinating and diploid species, is one of the most important annual legumes in the world due to its short growth period, high nutritional value, and multiple usages as hay, grain, silage, and green manure. The available simple sequence repeat (SSR) markers for common vetch, however, are insufficient to meet the developing demand for genetic and molecular research on this important species. Here, we aimed to develop and characterise several polymorphic EST-SSR markers from the vetch Illumina transcriptome. A total number of 1,071 potential EST-SSR markers were identified from 1025 unigenes whose lengths were greater than 1,000 bp, and 450 primer pairs were then designed and synthesized. Finally, 95 polymorphic primer pairs were developed for the 10 common vetch accessions, which included 50 individuals. Among the 95 EST-SSR markers, the number of alleles ranged from three to 13, and the polymorphism information content values ranged from 0.09 to 0.98. The observed heterozygosity values ranged from 0.00 to 1.00, and the expected heterozygosity values ranged from 0.11 to 0.98. These 95 EST-SSR markers developed from the vetch Illumina transcriptome could greatly promote the development of genetic and molecular breeding studies pertaining to in this species.

## 1. Introduction

The common vetch (*Vicia sativa* subsp. *sativa*) is an important forage legume crop that is commonly used as green manure, pasture, silage, and hay. The nutritional potential of its seeds is universally recognised, as they contain high levels of protein, starch, and oil [1,2]. The vetch also fixes atmospheric nitrogen through its symbiotic relationship with rhizobia. This species is a diploid with 2n = 2*x* = 12 chromosomes and a relatively small genome (2,205 Mb) compared with other *Vicia* species [3,4].

The development and use of codominant molecular markers has increased remarkably in the last decade. Codominant markers, which are locus-specific and multi-allelic, have applications in genetic diversity studies, cultivar identification, evolution, linkage mapping, QTL mapping, comparative genomics, and marker-assisted selection breeding. Traditional approaches based on probe hybridizaiton (containing repeated motifs) against genomic or cDNA libraries followed by DNA sequencing for the development of SSR markers are time-consuming and resource-intensive [5]. In the last few years, emphasis has shifted towards the development of SSR markers from the transcribed regions of the genome. Traditional approaches to the development of SSR markers are time-consuming and resource-intensive. There are two prominent advantages to using expressed transcripts, rather than genomic sequences, as molecular markers. First, expressed sequence tag (EST) derived markers are more likely embedded in functional gene sequences, which make them act as “functional genetic markers” for rapidly establishing marker-trait linkages and to identify genes quantitative trait loci (QTLs) for traits of agricultural importance in crop plants [6]. Therefore, EST-SSRs can provide opportunities for gene discovery and enhance the role of genetic markers by assaying variation in transcribed and known-function genes. Second, EST-SSR markers are likely to be more highly conserved and may be more transferable between closely related species compared with the markers derived from genomic sequences. Third, ESTs that show homology mapping and can be useful for aligning genome linkage maps across distantly related species for comparative analysis [7]. EST-SSR markers may also lead to direct gene tagging for QTL mapping of agronomically important traits, as well as increase the efficiency of marker-assisted selection [8]. Therefore, the development and characterisation of EST-SSR markers has become quite extensive in a wide range of plant species [1,9,10,11,12,13,14,15,16].

Recently developed high throughput sequencing technologies (*i.e.*, next-generation sequencing) performed on, for example, the Illumina Genome Analyser or the Roche/454 Genome Sequencer FLX Instrument, are powerful and cost-efficient tools for use on non-model organisms, especially in the development of EST-SSR markers [1,9,10,11,12,13,14,15,16]. During the past several years, next-generation sequencing for non-model organisms was largely confined to the Roche/454 instrument due to its longer reads [1,8,9,14]; however, several recent studies have demonstrated the feasibility of both 454 and Illumina technologies for the isolation of SSRs or EST-SSRs [1,9,10,11,12,13,14,15,16]. Due to Illumina’s high coverage and low cost, it is widely used in transcriptome sequencing. Using 454 pyrosequencing technology, 65 and 49 polymorphic EST-SSR markers have been developed in *V. sativa* subsp. *sativa* and *V. sativa* subsp. *nigra*, respectively [1,9]. Compared with some other plants (e.g., peanut, for which 1,281 polymorphic EST-SSR markers are available [17]), this number is low, the existing number of EST-SSR markers for common vetch is insufficient to meet the developing demand for genetic and molecular research on this plant.

In our previous study, we sequenced common vetch transcriptomes using Illumina technologies with NCBI accession No. GSE35437 [13]. Here, 1071 potential EST-SSRs were identified from 1025 unigenes whose lengths were greater than 1000 bp. By exploiting the Illumina sequencing databases of common vetch, we aimed to develop and characterise 95 novel polymorphic EST-SSR markers to promote studies on molecular diversity and breeding programs of this species.

## 2. Results and Discussion

*De novo* assembly of the vetch transcriptome using Illumina paired-end technology produced 43,973,369 raw sequence reads. All high-quality reads were assembled using the Trinity program [12], yielding 44,582 unigenes. We screened EST-SSR loci only from unigenes with lengths greater than 1,000 bp. Overall, 1,071 unigenes containing EST-SSR loci were identified and analysed. As shown in Table 1, we found that the most highly represented repeat number of potential EST-SSR loci was five, which accounted for 63.59% (681), followed by six (23.90%; 256), and seven (7.00%; 75). Di- to penta-nucleotide motifs were further analysed to determine the number of repeat units. A total of 41 potential EST-SSRs contained more than 13 repeat units, and all of the motifs were tri-nucleotide repeats. EST-SSRs are one of the most popular marker systems, consisting of varying numbers of tandem repeated di-, tri-, or tetra-nucleotide DNA motifs. As indicated in Table 1, the tri-nucleotide repeats were the most abundant type (97.20%; 1041), followed by di- (2.15%; 23), penta- (0.56%; 6), and tetra-nucleotide (0.09%; 1) repeats. Previous researches have shown that SSR occurrence in coding regions seems to be limited by non-perturbation of the reading frame, and the tri- and hexa-nucleotide repeats are dominant in protein-coding exons of all taxa. Such dominance of triplets over other repeats in coding regions may be explained on the basis of the suppression of non-trimeric SSRs in coding regions, possibly caused by frameshift mutations [18,19]. The relative proportions of EST-SSR motif types observed in vetch were similar to those seen in other plant species, such as Ma bamboo (*Dendrocalamus latiflorus*) [11] and alfalfa (*Medicago sativa*) [12].

**Table 1:** Length distribution of EST-SSR markers based on the number of repeat units.

No. of Repeat Units	Di-	Tri-	Tetra-	Penta-	Total
3	0	1	0	0	1
4	0	0	0	0	0
5	0	679	0	2	681
6	12	243	0	1	256
7	5	70	0	0	75
8	1	7	0	0	8
9	3	0	0	0	3
10	1	0	0	0	1
11	1	0	0	2	3
12	0	0	1	1	2
≥13	0	41	0	0	41
Total	23	1,041	1	6	1,071

Based on the sequences containing EST-SSRs, 450 pairs of EST-SSR primers were designed and synthesised. Five individuals from five accessions, including PI 179113, PI 181829, PI 187009, PI 201946, and PI 206392 (Table 2), were used for PCR amplification. Of the 450 primer pairs, 357 pairs were able to amplify PCR products from vetch genomic DNA, while 93 primer pairs failed to amplify PCR products at various annealing temperatures and Mg^2+^ concentrations, possibly due to the amplification of genomic DNA, the location of the primer across splice sites, large introns, chimeric primer or poor-quality sequences, and were thus excluded from further analysis. Among the 357 successful primer pairs, 115 PCR products showed the expected sizes, and 124 primer pairs generated PCR products that were larger than expected, indicating the likely presence of an intron within the amplicons. Additionally, the other 118 primer pairs were smaller than expected, indicating either the occurrence of deletions within the genomic sequences, a lack of specificity, or the possibility of assembly errors [12]. Of the 115 primer pairs that amplified PCR products with the expected sizes, 20 PCR products presented only one band, which may be a result of either the primer design or the homozygosity of the loci in alfalfa germplasm. There were 95 PCR amplifications that consistently resulted in more than one band among the 10 vetch accessions and 50 individual plants, which could be attributed to the high diversity of these 95 loci in regards to the 50 individuals.

**Table 2:** List of the common vetch (*Vicia sativa* subsp. *sativa*) accessions. Lanjian 3 cultivar comes from China.

No.	Accession No.	Origin
1	PI 179113	Turkey
2	PI 181828	Lebanon
3	PI 181829	Syria
4	PI 187009	Hungary
5	PI 193685	Belgium
6	PI 201946	Israel
7	PI 202524	Morocco
8	PI 206392	Cyprus
9	PI 206598	Greece
10	Lanjian 3	China

To determine whether the 95 EST-SSR markers developed in this study were novel, we searched the primer sequences of molecular markers previously reported in vetch against the target regions selected to design EST-SSR primers [1,9]. The BLAST results indicated that our 95 EST-SSR markers in vetch had not been previously reported. Information regarding the 95 novel EST-SSR primers is shown in Table 3 and Table S1.

Different locations of EST-SSR repeats within gene sequences may have different putative functions, for example, SSR variations in the 5'-UTR could regulate gene transcription and translation; SSR variations in the 3'-UTR could cause transcription slippage and produce expanded mRNA; and SSR variations in the coding regions should be subjected to much stronger selective pressure than those in other regions [10]. In the present study, 93 EST-SSR variations were found in the coding regions, while 2 were found in genes not associated with known proteins (Table S1).

**Table 3:** Characteristics of the 95 EST-SSR markers in vetch (*Vicia sativa* subsp. *sativa*).

Primer	Primer sequence (5'-3')	T_m_ (°C)	Size range (bp)	*N_A_*	*H_O_*	*H_E_*	*PIC*
VS-006	F:GTTGTTCTTAATGGTAAGTTGCTG R:TCCACCATTTTCAGTAGTAGCA	52	137–167	5	0.2	0.11	0.09
VS-009	F:CCCATAACAACTTCATCTTCATC R:GGAAGAGAGTAAGTGCATGTGTGT	54	123–153	7	0	0.69	0.63
VS-015	F:TTCGGGTGATGAAGAAGCT R:CAATTAAGCGTCTATATTCATCGG	54	164–194	7	0	0.81	0.78
VS-017	F:AACCTAGAATCCAAGACGACGA R:GAGGAAATGCGTCACAGTGAT	54	132–162	7	0	0.82	0.8
VS-018	F:AGAGAGAGAAGCTGCGATGTT R:TATGACTGTCTGGCTTTCTTGA	52	149–179	9	0.08	0.82	0.8
VS-020	F:TTTGAAGCAGCTTGTTGATGC R:TAACAAACAATTAGAGTCCAAGG	55	153–183	5	0.02	0.74	0.7
VS-025	F:TTGTTCGAATCATAATCACCG R:AAGAAGAGGATGATGAAGGAGA	52	168–204	6	0.05	0.25	0.24
VS-027	F:TCAGGTATCAAACTGGTTCATAAA R:TTGTGGTTGTGGTGGTGG	52	172–220	13	0.1	0.89	0.88
VS-029	F:GAACTAAGAATGGGAGGAGAAGA R:TCCGAATCCTCCCTGATGA	55	171–219	7	0	0.83	0.81
VS-030	F:AACCAAGACCACGATTCATC R:GACGCTCGGGTGTCTTTACATTT	58	181–211	5	0	0.66	0.59
VS-032	F:AGCCCCCTTCTATTGACC R:CCAGGAGGTATGTTTGCATT	54	167–227	7	0	0.69	0.65
VS-044	F:GAAGAAGCCATACAAGGACCTA R:ATGGGCAATCTAGTGGTGGTAA	55	136–166	7	1	0.83	0.81
VS-048	F:TGGGAAGTTGCTGGAGTTCT R:AGAATGAAATGCTCCTGCA	53	150–190	7	0.72	0.77	0.74
VS-053	F:AGTGATAGCGGCAGTGGCA R:ATAATCAATCCAAATTCTCTGGTC	56	136–166	7	0	0.82	0.79
VS-057	F:ACCCAAACAAGAGAACAAAGCATG R:CTTGCACCCATTTTTCTCTTGG	58	175–205	7	0	0.74	0.7
VS-060	F:AAGAGAACTCATTGCCCAGT R:CTTTACCTCACCCCTTTCACCTT	56	195–215	5	0	0.73	0.69
VS-063	F:TCAATCAGAAGCGACGTAAACG R:TAACAGCTCAGCCGTGCCT	56	168–198	5	0	0.53	0.5
VS-065	F:GGAGATTTATGTGTATCATGGTCT R:CCAAATTCCTTCAACTAAAAGAGG	54	184–214	7	0	0.78	0.75
VS-068	F:AGCATCCTTAGGAGAGGAATCC R:GCCTCGTGTTAGGGGACAGTTT	58	133–163	7	0	0.79	0.76
VS-075	F:GTCAACAGAAGGAACCTCGCAT R:GGAGCAAAAACATAAGCTAGGG	57	125–161	4	0.04	0.72	0.67
VS-086	F:CCATGATTAACTGAACCGCCTA R:ATCTGGAGGAGGAAAGGGA	55	141–171	7	0	0.82	0.8
VS-113	F:GACAGAATTGGTGATGCTAATGG R:TTTGTGCTTGAACTAGACGTACC	55	170–206	7	0	0.59	0.57
VS-115	F:TTTCCAAGATGACAGAATTGGTG R:AGACGTACCATTCACAGCATTT	55	166–202	7	0	0.68	0.66
VS-128	F:TTCAAGAGCGATTCGACGAT R:GCTTTTGGAGGGTATGGCTGTTT	59	184–214	5	0.04	0.76	0.72
VS-131	F:AAGTCTGGTCGGTAAAGGAACCT R:GAATGAGAAAACATGGCAAAGT	56	101–134	7	0	0.6	0.58
VS-134	F:TTCCCATCAAATGCAAGGTG R:CGCAAATAACTATCGTCTCTGAC	55	157–187	5	0.93	0.76	0.72
VS-138	F:CTTCTTCGGATTTACGGAGAGTGA R:ATGGCTGCTTCGGGTATC	57	154–190	4	0	0.55	0.45
VS-139	F:ACCACCCTTTTTCTTGAGCAG R:ATGGCAGTCGTGAGAGCTTT	54	155–185	7	0.06	0.76	0.72
VS-140	F:CTTTTTCTTCAACAGGCTTCCA R:GTAATAGAACGAGAAGAATCATT	55	168–198	7	0.08	0.78	0.74
VS-142	F:ATGCCCGACTCTTCAAGAAGTTT R:TACAATGCATAGGAGAGGAGACCT	57	160–202	7	0	0.65	0.59
VS-147	F:ACGGCTCGATGGACAGTAGTT R:TCAGTGTTTCTAAGGTTTTGCAT	55	172–208	11	0.48	0.62	0.55
VS-168	F:GACGACCTCCTTGACTTCTC R:CGGTGGAATTGGAGTTACTA	56	124–154	5	0.83	0.67	0.61
VS-169	F:GACGACCTCCTTGACTTCTC R:CGGTGGAATTGGAGTTACTA	56	124–154	7	0	0.78	0.75
VS-175	F:CTTTCCCCAAATCGAGTATC R:ACCTAGGTTGTGAGCTTGG	56	120–140	3	0.12	0.67	0.61
VS-183	F:TACCAACCTTGGCAGTTACA R:AAGGTGGAGATGTCCGATTA	57	143–173	4	0.89	0.75	0.71
VS-204	F:GGTTCCACAAACGACAATAC R:GTTCCCTCAACATCCAAATC	56	125–155	9	0.01	0.85	0.83
VS-206	F:TCACGAAGGAACTGATCAAC R:CTTCCACCAAAGATTCCAAG	57	124–160	6	0.02	0.79	0.76
VS-207	F:TCACGAAGGAACTGATCAAC R:CTTCCACCAAAGATTCCAAG	57	124–160	5	0	0.68	0.64
VS-251	F:ATTTCTTTAGAGCGGTGGAG F:CGAGCCATCAACAAACTC	56	115–151	12	0.07	0.98	0.98
VS-252	F:CTATGGTTATGAGCGTCCTG R:TAGTTCTTGCGATGGTGACT	55	141–171	9	0.25	0.69	0.67
VS-255	F:ATCATCCCCATCATAACCAC R:AGTTGCTGGGGTTCTAGGT	56	124–154	4	0.02	0.67	0.6
VS-257	F:TACTCCGTGTGGTGAAGTTT R:GGAGGCGGAGAGTAATAAGT	55	135–165	8	0	0.79	0.76
VS-258	F:CTCATCATGCACCTCAGATT R:GACTGATGCTGAAAAAGCAC	56	145–175	4	0	0.66	0.6
VS-259	F:GGAGGGATGTTGAAGTTTCT R:CAACCTTTGTTCAAGCTGAC	55	160–190	3	0.02	0.61	0.53
VS-267	F:GTGCAGAGAAATGCAAAGAG R:ACCACCACCACCTTGATAA	56	137–190	7	0.06	0.79	0.76
VS-272	F:GGAACTTGTCGATGTGATTG R:AAGATGAAGAAGACGGTGGT	56	124–154	4	0	0.72	0.67
VS-274	F:CATGAAGGAGTCAAAGGACA R:TAGCTCAAACTGCCTCAAAG	56	141–169	5	0.1	0.64	0.59
VS-276	F:CTCCGAAACATGGTTCATC R:TTCTCACTCTCACACTTTGC	56	146–176	7	0.96	0.86	0.84
VS-279	F:GATTGCCAGATATGCATGAG R:GCCGGGTTAAAGAGATTGT	56	116–146	9	0.08	0.94	0.94
VS-280	F:GATTGCCAGATATGCATGAG R:GCCGGGTTAAAGAGATTGT	56	116–146	3	0.96	0.5	0.38
VS-282	F:CGGAATATCAGAACTCAACG R:GATTGATGGTGAGGATGAGA	55	133–175	4	0	0.7	0.65
VS-286	F:CGTTAGCGGTATTTGTGGTA R:GAAGATACCTTGACCCTGCT	56	126–168	5	0	0.56	0.53
VS-292	F:AGATGATTGTGAGGAGACCA R:CTGTTGAGCACACTGTACC	55	127–157	7	0	0.81	0.78
VS-293	F:TCCACACTCAGTCTTCGTTT R:GCTCTCATCACAATCTGTCC	55	128–164	8	0.89	0.83	0.81
VS-295	F:ACACCACCAAGTGATCAAAG R:GGTAACCGTAGATGCTGAGA	55	133–163	7	0	0.81	0.78
VS-296	F:CAAAATCACCACTCCCACTA R:CTGTGACAAGGTTGTTGTTG	56	119–149	4	0.34	0.69	0.63
VS-297	F:TCATCACCCTGAGTATGACC R:CAACGTAAGGAACACTTGCT	55	137–167	4	0	0.66	0.6
VS-302	F:ACAACACCTCCCGTATCTTC R:CACTGTTTGTTGGGGAGTAA	56	123–159	5	0	0.77	0.73
VS-303	F:TGGCTCATATGGTGGTAATC R:GCATCTGTTCCATAACTTGC	55	126–156	10	0	0.78	0.75
VS-304	F:CAGTTGGGTCTTGTTTGTCT R:AAGCTCTCCTCATCAAAACC	56	136–166	6	0.25	0.76	0.72
VS-305	F:CTTTGCCTGTTCATCTTCTG R:GAACCTTGTTTCTTCCAAGC	56	173–203	5	0.18	0.78	0.74
VS-308	F:GAGTCTCGCTTCTCCATCTT R:GGGAGAGGGTATTTTGGTAA	56	123–153	7	0	0.78	0.76
VS-314	F:TCTGGGAGTAATTCACATGG R:GAGAAAGAAAACGCAGAAGG	56	150–192	7	0	0.75	0.71
VS-315	F:GCAAAGGTGTGAGAGTGAGA R:CTTCTGTTGTCGTGCAATG	56	151–181	5	0	0.65	0.57
VS-317	F:TGGAAGCACAGAAGATGAAG R:CTGATGTTGGTGACTTTGGT	56	131–161	9	0.16	0.74	0.7
VS-326	F:CCAAATGGAGGACCTATGAT R:GAGGATGAATTTGGAGCTGT	56	124–154	11	0	0.83	0.81
VS-333	F:CCTTCACGTCTTCATACCAA R:ACCTGATTCAAGTTCAGTGG	56	108–138	7	0	0.81	0.79
VS-343	F:TTGGAGTAGCATTCGATGTC R:GGGTCTGGTTGTTGAAGTTG	58	165–195	5	0	0.64	0.57
VS-358	F:GAGAAAGAGGTGGGTTTTTC R:CCGCTAGTACCAAACCCTAT	55	142–172	7	0	0.81	0.78
VS-363	F:CCTCTCATCCGTAGGATTTT R:GAGGTGGATTCCGGTAAAG	57	129–159	5	0	0.78	0.75
VS-373	F:GTGATTTCAACCACCAACAC R:GAGGTGGAGGGTATGAGTTT	56	126–162	5	0	0.76	0.72
VS-378	F:AGGTTCAATGCATCACTCC R:CCAACAACAGCAACAACAG	56	129–165	7	0	0.85	0.83
VS-379	F:TGATGGAGTTGGAGAAGATG R:TCCGCAGAAGTATCAGTGTC	56	136–166	9	0.44	0.82	0.8
VS-382	F:GGTCACGATTATCTCAACCA R:GTACCGGAGGAAGTGAAAAC	56	139–154	7	0	0.81	0.78
VS-406	F:GTTTGCAGCCATAGGAGGT R:CACCATCACAAGCTCCATT	57	125–155	5	0	0.77	0.73
VS-408	F:ACTTCCCCCAACTCTACAAA R:GGAAAAAGTCCAGACGAGAC	56	133–169	7	0	0.77	0.74
VS-415	F:GGTCCTTTCCTTTGTTCTTC R:GGTAACGCACATAGAAGCTG	56	126–156	4	0	0.74	0.69
VS-417	F:CGTTCATCAAAGCTTCCTC R:CATAGTTAAAGGCAGGCTCA	55	139–169	7	0	0.76	0.71
VS-418	F:TAGCTATTGTGCCTTGGGTA R:CGATGCTTCATCTTCTTCTC	56	122–152	8	0	0.86	0.84
VS-422	F:GAGTTCAGAAACTTCCCAGT R:CACCACTCTCCTTTCCTCTT	55	144–186	8	0.02	0.79	0.76
VS-423	F:CTCCTGAGTCTTGGCAAAT R:GGTTTACCAAACCAGCTACA	55	124–161	7	0	0.79	0.76
VS-425	F:GAGGAAGCATAAGAGGCACT R:CTCACCATGGAAGATTTCAG	56	125–155	7	0	0.82	0.8
VS-426	F:TTAGCACACAACACAACAGC R:GATCATTGACTTGGAGCAGA	56	165–195	7	0	0.84	0.82
VS-428	F:CCGTTCTGAATTATGTAGCC R:CATTTGAACTCCTTCACTGC	55	135–165	9	0	0.66	0.59
VS-429	F:GAGACTGGACAGATTGTTGG R:ACAACCCTTCTCTTCTGCTT	55	146–182	9	0	0.87	0.86
VS-430	F:GTCATTCCCATGGTTCTCAT R:GTCAGTCACTCGACTGGAAA	57	125–155	7	0	0.62	0.6
VS-431	F:CAGGATAATGTTCTCCACCA R:GAGCTTCTTGGGATGGTTAT	55	147–183	4	0.02	0.67	0.61
VS-433	F:TACTTAGTTCGGCCGGTATT R:GGCCCACCTACATTGATACT	56	136–166	6	0.56	0.79	0.76
VS-437	F:ACAACAACATCACCCTTCAG R:TATCTGGACCAACTGATTGC	56	137–167	6	0	0.83	0.81
VS-439	F:AGCATCATTCAGGAGACAAG R:TGCTCCAGCTGATCTACTTC	55	139–169	7	0	0.81	0.78
VS-440	F:ATCCCTCAACCTTGATCTGT R:ATATTGGGATCTGGGTTCC	56	125–161	5	0	0.76	0.72
VS-441	F:CTTGGTTAGATTGCAACGAC R:ACGCGACAGTAGCATAGAAA	55	136–166	6	0	0.83	0.81
VS-442	F:CAACGGCTTAAAGAGAAGGT R:TACATCATAAACCGGTACGC	56	150–180	6	0	0.8	0.77
VS-445	F:AGAAGCGCAACAGTCTTGTA R:AGAAGACGCACTTTACCTGA	56	137–170	5	0.52	0.64	0.59
VS-447	F:GAGACCAAGGGAACTGAATC R:CAGATTGATTAGGCAGCAAG	56	157–187	9	0.21	0.85	0.83

Note: (*N_A_*), number of allele; *(H_O_*), observed heterozygosity; (*H_E_*), expected heterozygosity; (*PIC*), polymorphic information content; (T_m_), melting temperature.

**Figure 1:**
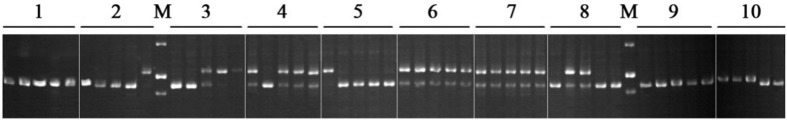
EST-SSR marker variations at the VS-006 locus of 10 vetch accessions. Each accession includes five individual plants. The corresponding detailed information from the 10 vetch accessions is displayed in Table 2. The letter “M” denotes the molecular markers, which are 300 bp, 200 bp, and 150 bp (top to bottom).

For the polymorphic 95 EST-SSR loci, the number of alleles (*N_A_*) per locus varied widely among the markers (Table 3 and Figure 1) and ranged from 3 to 13, with an average of 6.49 alleles. The observed heterozygosity (*H_O_*) values ranged from 0.00 to 1.00, with an average of 0.12, and the expected heterozygosity (*H_E_*) values ranged from 0.11 to 0.98, with an average of 0.74. Finally, the polymorphic index content (*PIC*) values ranged from 0.09 to 0.98, with an average of 0.70. Similar observations were also made in previous studies of *V. sativa* subsp. *sativa*, for which the average *PIC* value was 0.59 [1], and of *V. sativa* subsp. *nigra*, for which the average *PIC* value was 0.59 [9].

**Figure 2:**
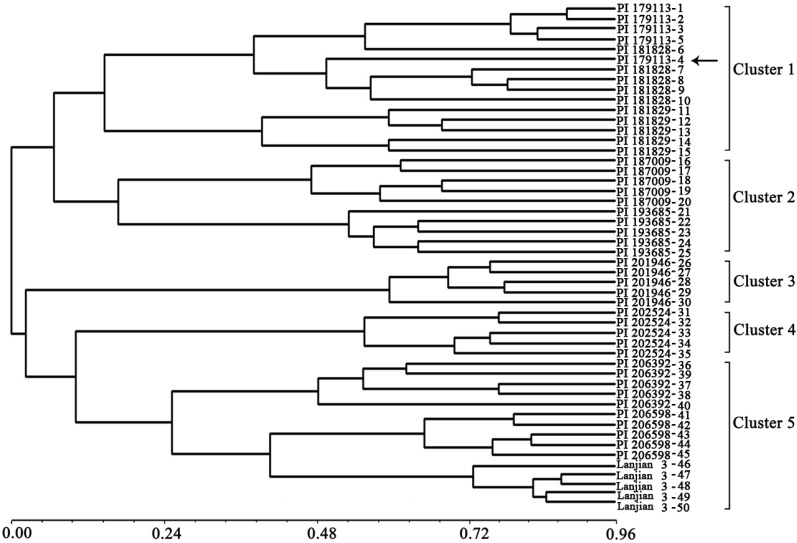
Dendrogram generated using NTSYS cluster analysis based on the genetic diversity of 10 vetch (*Vicia sativa* subsp. *sativa*) accessions.

The dendrogram showed that the 50 individual vetch plants fell into five distinct clusters (Figure 2). Accessions cluster 1 originated from West Asia, cluster 2 from Europe, cluster 3 from West Asia, cluster 4 from Africa, and cluster 5 from Asia and Europe. The individual plants of No. 40–45 a Greek accession were clustered with a Chinese vetch cultivar (No. 46–50), indicating that the two accessions may share some genetic background. Moreover, only individual plant No. 4 was not clustered with its own group, which may be a result of genetic variation within accessions. In a previous study, researchers constructed dendrograms of *V. sativa* subsp. *sativa* and *V. sativa* subsp. *nigra*, and found no clear relationship between the clustering pattern and geographical distance [1,9]. Our cluster result is similar to those reported in the two previous studies, suggesting that the use of a greater number of accessions from close geographical locations will be essential to verify our present conclusion in future studies.

## 3. Experimental

### 3.1. Plant Material

The common vetch seeds of 10 accessions (Table 2) were selected from the United States Department of Agriculture National Plant Germplasm System (NPGS). The common vetch seeds of 10 accessions (Table 2) were selected from the National Plant Germplasm System (NPGS). Seedlings were grown in a greenhouse at Lanzhou University in Lanzhou, China for approximately 45 days under a 16 h light/8 h dark cycle at 22 °C. Five individual plants from each of the 10 vetch accessions were used for polymorphism investigations of the selected EST-SSR markers. Genomic DNA was extracted from young leaves according to an established cetyltrimethylammonium bromide (CTAB) method [20], and DNA quantity and quality were assessed using a NanoDrop ND1000 instrument (Thermo Scientific, Wilmington, DE, USA).

### 3.2. Detection of EST-SSR Markers and Primer Design

The Illumina sequencing data for 44,582 unigenes had been previously deposited in the NCBI Gene Expression Omnibus with accession No. GSE35437 [13]. EST-SSRs were detected in the 44,582 vetch unigenes using the Simple Sequence Repeat Identification Tool program [12]. Only unigenes longer than 1000 bp were included in the EST-SSR detection. The parameters were adjusted to identify perfect di-, tri-, tetra-, and penta-nucleotide motifs with a minimum of 6, 5, 5, and 5 repeats, respectively. The EST-SSR primers were designed using Batch Primer3 [12].

The following parameters were used for primer design: (1) primer length between 18 and 24 bp, with 20 bp as the optimum; (2) PCR product size from 100 to 250 bp; (3) annealing temperature from 50 to 60 °C and with an optimum annealing temperature of 55 °C; (4) GC content between 45% and 55%, with 50% as the optimum.

### 3.3. PCR Amplification and Diversity Analysis

PCR amplification was performed in a 10 μL reaction volume containing 50 ng of genomic DNA, 1× PCR buffer, 2.0 mM MgCl_2_, 2.5 mM dNTPs, 4.0 µM each primer, and 0.8 U Taq polymerase (TaKaRa, Kyoto, Japan). The cycling parameters were 94 °C for 3 min and 38 cycles of the following: 94 °C for 35 s, optimal annealing temperature (Table 3) for 35 s, and 72 °C for 35 s, followed by a final extension at 72 °C for 7 min. The PCR products were subjected to electrophoresis on 8.0% non-denaturing polyacrylamide gels and then stained with ethidium bromide [12]. The band sizes were determined by comparison with the DL 500 DNA marker (TaKaRa, Kyoto, Japan). The indexes of *H_O_*, *H_E_*, and *PIC* were calculated as previously described [1]. Clusters analysis was performed to generate a dendrogram using the unweighted pair-group method with arithmetic mean (UPGMA) and Nei’s unbiased genetic distance with NTSYSPC 2.0 software package [21]. 

## 4. Conclusions

By exploiting the Illumina sequencing database, we developed 95 novel EST-SSR markers, which were then successfully used to investigate the genetic diversity among 10 vetch accessions. To date, this represents the largest known number of vetch SSR markers developed in a single study. These 95 EST-SSR markers with relatively high degrees of polymorphism could be applied to a range of studies involving genetic diversity, cultivar identification, evolution, linkage mapping, QTL mapping, comparative genomics, and marker-assisted selection breeding of common vetch.

## Supplementary Materials

Supplementary materials can be accessed at: http://www.mdpi.com/1420-3049/19/5/5777/s1.
