# Supplementary material for: Exploiting Illumina Sequencing for the Development of 95 Novel Polymorphic EST-SSR Markers in Common Vetch (Vicia sativa subsp. sativa)
Source: Molecules. 2014 May 5;19(5):5777–89. doi: 10.3390/molecules19055777 (PMC6271487; doi:10.3390/molecules19055777)
Supplement: Supplementary file 1 [file molecules-19-05777-s001.pdf]

Supplementary File

Table S1. Functional annotations of the 95 EST-SSR markers in common vetch (*Vicia sativa* subsp. *sativa*).

Primer	SSR motif	Nt-Score	Nt-Evalue	Nt-Annotation
VS-006	(AAG)5	807	0	*Medicago truncatula* Ribosomal RNA processing protein-like protein (MTR_4g133100) mRNA, complete cds
VS-009	(TCT)5	1287	0	*Medicago truncatula* clone mth2-11a6, complete sequence
VS-015	(CGA)5	2940	0	*Pisum sativum* mRNA for phospholipase C
VS-017	(CAC)5	809	0	*Medicago truncatula* YLP motif-containing protein (MTR_4g107890) mRNA, complete cds
VS-018	(GAT)6	2198	0	PREDICTED: *Glycine max* DEAH (Asp-Glu-Ala-His) box polypeptide 16, transcript variant 1 (DHX16), mRNA
VS-020	(TTG)5	884	0	*Pisum sativum* MADS box protein M3 mRNA, complete cds
VS-025	(AAC)6	93.7	2.00×10^{-16}–16	*Medicago truncatula* Ethylene-responsive transcription factor 1A (MTR_5g075570) mRNA, complete cds
VS-027	(AGG)8	1150	0	*Medicago truncatula* BEL1-like homeodomain protein (MTR_5g018860) mRNA, complete cds
VS-029	(AGG)8	236	2.00×10^{-16}–59	*Medicago truncatula* chromosome 5 clone mte1-84j2, COMPLETE SEQUENCE
VS-030	(GAT)5	4482	0	*Medicago truncatula* Inositol hexakisphosphate and diphosphoinositol-pentakisphosphate kinase (MTR_7g009060) mRNA, complete cds
VS-032	(CAA)5N(CAA)6	474	1.00×10^{-16}–131	*Medicago truncatula* chromosome 5 clone mth2-124f10, COMPLETE SEQUENCE
VS-044	(CCA)5	283	5.00×10^{-16}–74	*Pisum sativum* clone PsEXT5.5 root nodule extensin mRNA, complete cds
VS-048	(TAAC)5	543	1.00×10^{-16}–151	*Medicago truncatula* Protein kinase 2B (MTR_5g099130) mRNA, complete cds
VS-053	(TTG)5	952	0	*Medicago truncatula* WRKY transcription factor WRKY100630 mRNA, complete cds
VS-057	(GAA)5	1181	0	PREDICTED: *Glycine max* cyclic nucleotide-gated ion channel 4-like (LOC100796761), mRNA
VS-060	(GAA)5	2058	0	*Medicago truncatula* Bromodomain-containing protein (MTR_3g052530) mRNA, complete cds
VS-063	(TGT)5	930	0	*Medicago truncatula* Aluminum activated malate transporter (MTR_8g104120) mRNA, complete cds
VS-065	(TTC)5	848	0	*Medicago truncatula* Vicilin-like antimicrobial peptides 2-3 (MTR_2g093350) mRNA, complete cds
VS-068	(TCC)5	737	0	PREDICTED: *Glycine max* two-component response regulator-like APRR1-like (LOC100786522), mRNA
VS-075	(AGG)6	174	8.00×10^{-16}–41	PREDICTED: *Glycine max* uncharacterized protein LOC100787910 (LOC100787910), mRNA
VS-086	(TCC)5	--	--	--
VS-113	(TGA)6	2159	0	PREDICTED: *Glycine max* serine/threonine-protein phosphatase 6 regulatory subunit 1-like, transcript variant 1 (LOC100802276), mRNA

Table S1. *Cont.*

Primer	SSR motif	Nt-Score	Nt-Evalue	Nt-Annotation
VS-115	(TGA)6	438	1.00×10^{-16}–120	PREDICTED: *Glycine max* serine/threonine-protein phosphatase 6 regulatory subunit 3-like (LOC100805409), miscRNA
VS-128	(CAC)5	387	1.00×10^{-16}–104	PREDICTED: *Glycine max* cell number regulator 5-like (LOC100786186), mRNA
VS-131	(TCT)6	141	1.00×10^{-16}–30	*Medicago truncatula* clone mth2-144j8, complete sequence
VS-134	(CAA)5	1195	0	PREDICTED: *Glycine max* uncharacterized protein LOC100809022 (LOC100809022), mRNA
VS-138	(AGA)6	1645	0	*Medicago truncatula* Glutamate-1-semialdehyde 2,1-aminomutase (MTR_3g118070) mRNA, complete cds
VS-139	(TTC)5	2403	0	*Medicago truncatula* clone mth2-165n2, complete sequence
VS-140	(TTC)5	2296	0	*Medicago truncatula* clone mth2-165n2, complete sequence
VS-142	(ACC)7	190	1.00×10^{-16}–45	PREDICTED: *Glycine max* uncharacterized protein LOC100804987 (LOC100804987), mRNA
VS-147	(CAA)6	722	0	*Medicago truncatula* Two-component response regulator ARR18 (MTR_2g086450) mRNA, complete cds
VS-168	(GAA)5	375	1.00×10^{-16}–101	*Medicago truncatula* GATA transcription factor (MTR_3g117380) mRNA, complete cds
VS-169	(GAA)5	375	1.00×10^{-16}–101	*Medicago truncatula* GATA transcription factor (MTR_3g117380) mRNA, complete cds
VS-175	(ATC)5	1096	0	*Medicago truncatula* MPBQ/MSBQ methyltransferase (MTR_1g071110) mRNA, complete cds
VS-183	(CAC)5	--	--	--
VS-204	(GAG)5	741	0	*Medicago truncatula* chromosome 7 BAC clone mth2-7n16, complete sequence
VS-206	(GAA)6	957	0	*Medicago truncatula* R3H domain-containing protein (MTR_1g092900) mRNA, complete cds
VS-207	(GAA)6	932	0	*Medicago truncatula* R3H domain-containing protein (MTR_1g092900) mRNA, complete cds
VS-251	(GGA)6	91.7	6.00×10^{-16}–16	PREDICTED: *Glycine max* succinate dehydrogenase [ubiquinone] iron-sulfur subunit 2, mitochondrial-like (LOC100796630), mRNA
VS-252	(GAT)5	125	4.00×10^{-16}–26	*Medicago truncatula* chromosome 6 clone mth2-31d18, complete sequence
VS-255	(CAT)5	186	1.00×10^{-16}–44	*Lotus japonicus* cDNA, clone: LjFL1-014-AB12, HTC
VS-257	(GGT)5	151	1.00×10^{-16}–33	*Medicago truncatula* chromosome 2 BAC clone mth2-181i5, complete sequence
VS-258	(TTC)5	950	0	*P. sativum* mRNA for HMG1 protein
VS-259	(TTC)5	155	1.00×10^{-16}–34	PREDICTED: *Glycine max* uncharacterized protein LOC100527689 (LOC100527689), mRNA

Table S1. *Cont.*

Primer	SSR motif	Nt-Score	Nt-Evalue	Nt-Annotation
VS-267	(TAA)5N(GGT)5	549	1.00×10^{-16}–153	*Medicago truncatula* clone mth2-19b12, complete sequence
VS-272	(GAA)5	283	1.00×10^{-16}–73	*Medicago truncatula* clone mth2-53p19, complete sequence
VS-274	(TTA)5	246	4.00×10^{-16}–62	PREDICTED: *Glycine max* ethylene-responsive transcription factor ERF105-like (LOC100816416), mRNA
VS-276	(TGA)5	250	4.00×10^{-16}–63	*Medicago truncatula* clone mth2-11a6, complete sequence
VS-279	(GAT)5	85.7	1.00×10^{-16}–13	PREDICTED: *Glycine max* uncharacterized protein LOC100803890 (LOC100803890), mRNA
VS-280	(GAT)5	87.7	1.00×10^{-16}–14	*Medicago truncatula* hypothetical protein (MTR_080s0045) mRNA, complete cds
VS-282	(ATC)7	2365	0	*Pisum sativum* translation initiation factor mRNA, complete cds
VS-286	(GTT)7	184	3.00×10^{-16}–43	*Lotus japonicus* genomic DNA, clone: LjT02J13, TM2022, complete sequence
VS-292	(GGA)5	1515	0	*Medicago truncatula* DnaJ protein-like protein (MTR_8g006430) mRNA, complete cds
VS-293	(GTT)6	216	4.00×10^{-16}–53	PREDICTED: *Glycine max* homeobox-leucine zipper protein ATHB-6-like (LOC100777924), mRNA
VS-295	(AAC)5	135	4.00×10^{-16}–29	*Medicago truncatula* Solute carrier family 35 member E4 (MTR_4g101860) mRNA, complete cds
VS-296	(ACC)5	391	1.00×10^{-16}–105	*Medicago truncatula* ZF-HD homeobox protein (MTR_7g010300) mRNA, complete cds
VS-297	(TGA)5	1017	0	*Pisum sativum* mRNA for MADS-box transcription factor
VS-302	(ATG)6	1737	0	*Medicago truncatula* Transcription elongation factor A protein (MTR_3g108010) mRNA, complete cds
VS-303	(CTG)5	1572	0	*Medicago truncatula* HIV Tat-specific factor-like protein (MTR_3g031740) mRNA, complete cds
VS-304	(GTT)5	482	1.00×10^{-16}–133	*M. truncatula* DNA sequence from clone MTH2-18N13 on chromosome 3, complete sequence
VS-305	(TCT)5	176	4.00×10^{-16}–41	*Lotus japonicus* genomic DNA, chromosome 6, clone: LjT19B18, TM0139, complete sequence
VS-308	(GGC)5	172	7.00×10^{-16}–40	*M. truncatula* DNA sequence from clone MTH2-33P23 on chromosome 3, complete sequence
VS-314	(TCA)7	65.9	2.00×10^{-16}–08	*Glycine max* strain Williams 82 clone GM_WBa0065P04, complete sequence
VS-315	(AGA)5	214	2.00×10^{-16}–52	*M. truncatula* DNA sequence from clone MTH2-116J14 on chromosome 3, complete sequence
VS-317	(GTG)5	204	1.00×10^{-16}–49	*Medicago truncatula* chromosome 5 clone mth4-58a13, COMPLETE SEQUENCE
VS-326	(AAT)5	1304	0	*Medicago truncatula* BZIP transcription factor (MTR_2g099050) mRNA, complete cds
VS-333	(ACA)5	270	2.00×10^{-16}–69	*Glycine max* peptide methionine sulfoxide reductase B1, chloroplastic-like (LOC100798757), mRNA
VS-343	(CAA)5	167	7.00×10^{-16}–39	*Lotus japonicus* genomic DNA, chromosome 6, clone: LjT03K02, TM1763, complete sequence
VS-358	(GTG)5	121	2.00×10^{-16}–24	PREDICTED: *Glycine max* uncharacterized protein LOC100798129 (LOC100798129), mRNA

Table S1. *Cont.*

Primer	SSR motif	Nt-Score	Nt-Evalue	Nt-Annotation
VS-363	(CAT)5	575	1.00×10^{-16}–161	*Medicago truncatula* BHLH transcription factor (MTR_5g037250) mRNA, complete cds
VS-373	(CAG)6	545	1.00×10^{-16}–152	*Medicago truncatula* Squamosa promoter binding protein (MTR_8g005960) mRNA, complete cds
VS-378	(TGT)6	93.7	2.00×10^{-16}–16	PREDICTED: *Glycine max* ethylene-responsive transcription factor ERF053-like (LOC100799750), mRNA
VS-379	(TGA)5	521	1.00×10^{-16}–145	*Medicago truncatula* clone mth2-24i7, complete sequence
VS-382	(CAT)5	73.8	3.00×10^{-16}–10	*Medicago truncatula* chromosome 8 clone mth2-173b11, complete sequence
VS-406	(GGT)5	165	4.00×10^{-16}–38	*Lotus japonicus* cDNA, clone: LjFL1-003-BA12, HTC
VS-408	(TGG)6	799	0	*Medicago truncatula* Chloroplast processing peptidase (MTR_8g103470) mRNA, complete cds
VS-415	(CTT)5	125	4.00×10^{-16}–26	PREDICTED: *Glycine max* uncharacterized protein LOC100819460 (LOC100819460), mRNA
VS-417	(CAT)5	482	1.00×10^{-16}–133	PREDICTED: *Glycine max* uncharacterized protein LOC100799451 (LOC100799451), mRNA
VS-418	(AAC)5	105	8.00×10^{-16}–20	*Medicago truncatula* clone mth2-32h4, complete sequence
VS-422	(ATG)7	317	8.00×10^{-16}–84	*Medicago truncatula* WRKY transcription factor WRKY108715 mRNA, complete cds
VS-423	(TGT)6	373	1.00×10^{-16}–100	*Medicago truncatula* chromosome 5 clone mte1-7c20, COMPLETE SEQUENCE
VS-425	(TGC)5	143	2.00×10^{-16}–31	*Lotus japonicus* genomic DNA, chromosome 4, clone: LjT23O19, TM0297b, complete sequence
VS-426	(TCA)5	971	0	*Medicago truncatula* ABC transporter I family member (MTR_8g101390) mRNA, complete cds
VS-428	(AGC)5	611	1.00×10^{-16}–172	*Lotus japonicus* cDNA, clone: LjFL1-015-CG02, HTC
VS-429	(TTG)6	238	4.00×10^{-16}–60	PREDICTED: *Glycine max* uncharacterized protein LOC100790563 (LOC100790563), mRNA
VS-430	(CCG)5	383	1.00×10^{-16}–103	*Medicago truncatula* F-box family protein (MTR_3g099300) mRNA, complete cds
VS-431	(TTC)6	313	3.00×10^{-16}–82	*Lotus japonicus* genomic DNA, clone: LjT24M05, TM1610, complete sequence
VS-433	(TGG)5	307	2.00×10^{-16}–80	PREDICTED: *Glycine max* uncharacterized protein LOC100799791 (LOC100799791), mRNA
VS-437	(GCA)5	698	0	*Lotus japonicus* cDNA, clone: LjFL3-067-AH02, HTC
VS-439	(CAA)5	184	6.00×10^{-16}–44	*Medicago truncatula* chromosome 8 clone mth2-130n5, complete sequence
VS-440	(TTG)6	343	2.00×10^{-16}–91	*Medicago truncatula* Zinc finger protein CONSTANS-like protein (MTR_1g023260) mRNA, complete cds
VS-441	(AAC)5	87.7	3.00×10^{-16}–14	PREDICTED: *Glycine max* U-box domain-containing protein 18-like (LOC100810963), mRNA
VS-442	(AGA)5	1358	0	*Medicago truncatula* CONSTANS-like zinc finger protein (MTR_8g104190) mRNA, complete cds
VS-445	(TTG)6	916	0	PREDICTED: *Glycine max* probable esterase At1g33990-like (LOC100784567), mRNA
VS-447	(AAC)5	1164	0	*Medicago truncatul*a Pantothenate kinase (MTR_3g077740) mRNA, complete cds
